# Multimodality Imaging of Warthin’s Tumor: PET/CT, Scintigraphy, MRI, and CT

**DOI:** 10.3390/diagnostics15212666

**Published:** 2025-10-22

**Authors:** Miju Cheon, Hyunkyung Yi, Injoong Kim

**Affiliations:** 1 Department of Nuclear Medicine, Veterans Health Service Medical Center, Seoul 05368, Republic of Korea; 2 Department of Radiology, Veterans Health Service Medical Center, Seoul 05368, Republic of Korea

**Keywords:** Warthin’s tumor, parotid gland, [^18^F]FDG PET/CT, salivary scintigraphy, multimodality imaging

## Abstract

Warthin’s tumor is a benign salivary gland neoplasm that can exhibit intense FDG uptake, potentially mimicking malignant lesions on oncologic imaging. We report a case of a 78-year-old man undergoing staging for suspected lung cancer, in whom a hypermetabolic lesion was incidentally detected in the left parotid gland on [^18^F]FDG PET/CT. Correlation with prior salivary scintigraphy, MRI, and CT supported the likelihood of Warthin’s tumor, which was subsequently confirmed by fine-needle aspiration cytology. This case illustrates how multimodality imaging can provide complementary diagnostic information that helps characterize the parotid lesion, but not replace cytologic confirmation. Recognition of characteristic imaging features, an understanding of each modality’s diagnostic strengths and limitations, and cytologic confirmation when indicated are essential to avoid misinterpretation and optimize patient management.

**Figure 1:**
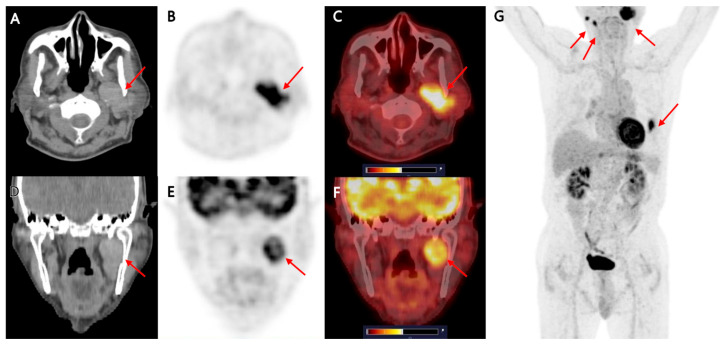
A 78-year-old man with long-standing interstitial lung disease was suspected of having lung cancer on his routine chest computed tomography (CT). He initially underwent [^18^F]FDG positron emission tomography (PET)/CT, chest CT, and brain magnetic resonance imaging (MRI) as part of a staging work-up for a suspected primary lung cancer. As part of the staging work-up, [^18^F]FDG PET/CT was performed first. [^18^F]FDG PET/CT demonstrated a 22 mm FDG-avid consolidative nodule (SUVmax 12.18) in the lingular division of the left lung, suggesting lung cancer ((**G**); red arrow). Besides the abnormal FDG uptake of the lesion in the left lung, axial and coronal CT ((**A**,**D**); red arrow), PET ((**B**,**E**); red arrow), and fused PET/CT images ((**C**,**F**); red arrow) of [^18^F]FDG PET/CT show a 4.0 × 1.7 × 3.2 cm, well-marginated mass in the left level II cervical region, showing iso- to hyperdense appearance on corresponding non-contrast CT and marked FDG avidity (SUVmax 10.53). The lesion appeared to arise from the inner portion of the left parotid gland, protruding into the level II neck space without definite evidence of surrounding tissue invasion. In the PET/CT staging for suspected lung cancer, the lesion in the left parotid gland was incidentally detected, in other words, discovered during imaging performed for another indication. Ultrasonography with or without fine-needle aspiration cytology (FNAC) is the standard and minimally invasive approach for evaluating parotid tumors. For the left parotid mass, although we considered Warthin’s tumor as a first differential diagnosis, we still had to exclude the possibility of a malignant parotid tumor or metastatic lymphadenopathy. Therefore, to increase confidence in the interpretation of the PET/CT finding, we tried to identify the temporal changes in the lesion first. Additionally, we observed two nodular lesions, approximately 5 mm in size, with increased FDG uptake in the right parotid gland on the maximum intensity projection image (**G**), with SUVmax values of 9.7 and 9.1, respectively. Although the right parotid lesions were not further investigated because they were clinically asymptomatic, and given their imaging appearance, they were considered more likely benign, possibly an additional Warthin’s tumor.

**Figure 2:**
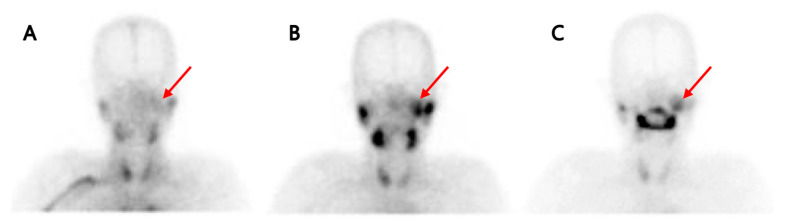
To assess the lesion’s temporal change, we reviewed past images and identified salivary scintigraphy images from approximately one year ago with dry mouth symptoms. Sequential pertechnetate salivary gland scintigraphy images at 30 min (**B**) revealed a lesion (red arrow) with strongly increased uptake just medial to the left parotid gland in the 30 min image, which was not clearly visible in the 1 min image (**A**). Also, the lesion showed persistent retention after stimulant administration (**C**). Retrospectively, this lesion matched the location and shape of the lesion seen in the [^18^F]FDG PET/CT. Considering the [^18^F]FDG PET/CT and the salivary gland scintigraphy images taken one year prior, although the size could not be precisely compared, there was no significant change in size, the location and shape were correlated, and considering the findings in the two images, we could first consider the lesion in the left parotid gland as Warthin’s tumor. At that time, the left parotid lesion was not clinically suspected and overlooked, and the clinician did not regard it as a mass requiring further imaging or biopsy. Therefore, no further diagnostic work-up, such as ultrasonography or FNAC, was performed then. In some situations, such as incidental findings in asymptomatic patients, initial management decisions may be guided by imaging features and clinical presentation. Importantly, a watch-and-wait policy is acceptable only in asymptomatic patients without facial nerve dysfunction, dysphagia, or other functional compromise. Careful follow-up with serial imaging is therefore essential in such cases. While salivary gland scintigraphy is not routinely used to distinguish benign from malignant parotid tumors, specific characteristic patterns, such as intense uptake with persistent tracer retention post-stimulation, are strongly suggestive of Warthin’s tumor. Salivary gland scintigraphy remains a clinically useful tool for identifying parotid gland tumors and for assessing gland function. However, its standalone accuracy for reliably distinguishing between benign and malignant parotid tumors is limited and benefits from multimodal correlation. To our knowledge, there is limited published data on its sensitivity and specificity in differentiating benign from malignant parotid lesions [1].

**Figure 3:**
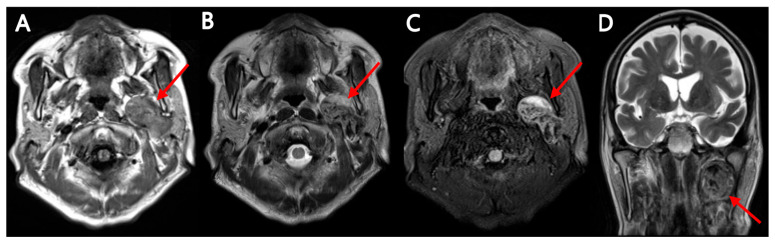
Brain-screen MRI, which was performed to determine the stage of the lung cancer, also revealed a well-defined, approximately 4.4 cm-sized lobulating mass (red arrow) in the left parotid space. On the available brain-screen MRI sequences, the lesion showed a low to intermediate T1 signal intensity (**A**) and heterogeneous intermediate to high T2 signal intensity (**B**,**D**). The fluid-attenuated inversion recovery (FLAIR) image showed heterogeneous low to high signal intensity (**C**). We acknowledge that this MRI was a brain screening study rather than a dedicated parotid/neck protocol. Brain MRI may detect large parotid masses that lie within the imaged field; however, it lacks some small-field-of-view sequences, such as high-resolution fat-suppressed T2 and dynamic contrast perfusion, as well as coil optimization used in dedicated parotid/neck MRI. Therefore, while the brain MRI supported the presence of a sizable, well-circumscribed mass without overt invasive features, a dedicated parotid/neck MRI or targeted ultrasound would be preferable when fine tissue characterization is required.

**Figure 4:**
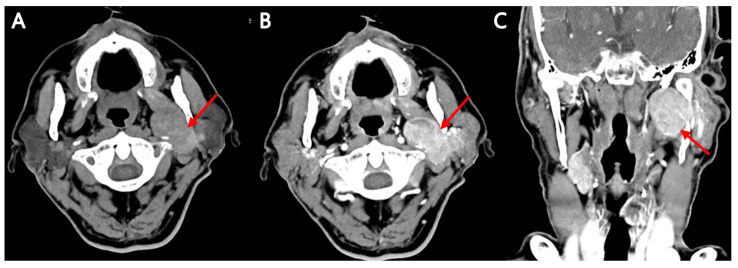
A dedicated contrast-enhanced neck CT was subsequently performed. Axial non-enhanced computed tomography images (**A**) in soft tissue window and contrast-enhanced axial (**B**) and coronal (**C**) images also demonstrated a 4.8 cm lobulating, heterogeneously enhancing mass involving the left parotid–parapharyngeal space, with an internal cystic component (red arrow). Taking these findings into account, we could consider Warthin’s tumor rather than metastatic lymphadenopathy or malignant parotid tumor. The contrast-enhanced neck CT provided higher spatial resolution and more detailed information on lesion architecture, enhancement patterns, internal cystic components, and relationships to adjacent structures than the low-dose CT component of the preceding PET/CT. This information is useful for procedural planning and helps guide whether image-guided FNAC is needed and how it should be performed. In this case, the cross-modal concordance of PET/CT, scintigraphy, MRI, and dedicated CT supported a working diagnosis of Warthin’s tumor. Finally, fine-needle aspiration was performed, and cytology confirmed the diagnosis. Warthin’s tumor is the second most common benign salivary gland tumor, typically arising in the parotid gland of older adults, with a strong association with smoking [2]. Although benign, it frequently demonstrates high FDG avidity, as demonstrated in this case, which may mimic malignancy and confound staging in oncologic patients. According to Kowalewski et al. SUVmax for the Warthin’s tumors ranged from 3.6 to 26.8, with an average SUVmax of 10.1. Also, Warthin’s tumors exhibit significant and variable FDG accumulation, exceeding expectations and mimicking high-grade malignancies. In cases of positive FDG uptake in periparotid, perimandibular, and upper jugular areas, as seen in this case, needle aspiration is recommended to avoid misinterpretation [3]. On CT and MRI, Warthin’s tumor is usually seen as a well-circumscribed lesion with a heterogeneous solid-cystic structure. With contrast enhancement, we can see early, intense enhancement and rapid washout [4,5]. Additionally, although MRI and CT have reasonably high accuracy in differentiating benign from malignant parotid tumors in many series, their performance varies by technique and lesion type [6,7]. Because of its vascular and cystic elements, Warthin’s tumor may mimic malignant lesions on these imaging studies. Multiparametric MRI (conventional sequences plus diffusion and perfusion metrics) and advanced methods such as radiomics have reported diagnostic accuracies commonly in the 80–90% range and AUCs > 0.8 in several studies [8,9,10]. Nevertheless, overlap between benign and malignant imaging features can occur, and no single imaging modality is sufficient on its own. Therefore, imaging should be considered complementary to clinical assessment and cytology, and dedicated imaging protocols (neck/parotid MRI or contrast neck CT) are preferred when management decisions require detailed local staging or tissue characterization. Salivary gland scintigraphy plays a complementary role and, in some cases, its distinctive appearance is used as a problem-solving tool. The lesion typically shows focal increased tracer uptake and does not wash out with stimulation. In contrast to other benign parotid tumors, pleomorphic adenomas show a cold area on pertechnetate salivary scintigraphy. The reason for these findings is that Warthin’s tumor is characterized by a rich lymphoid stroma and neoplastic epithelial cells, and it is known that ^99m^Tc-pertechnetate is not well excreted but trapped [11]. Nevertheless, so far, no non-invasive imaging test alone reliably rules out malignancy in all cases, especially in lesions with atypical features, deep location, or suspicious behavior. FNAC remains a key confirmatory tool. In the present case, it demonstrated intense FDG uptake, mimicking a suspicion of metastatic disease or a malignant parotid tumor. Although Warthin’s tumor was also the first differential diagnosis considered in CT and MRI, the possibility of a malignant lesion could not be completely ruled out. In this case, in particular, more attention should be paid to the differential diagnosis because the location was in the peripharyngeal area. However, the correlation with salivary scintigraphy, together with the lack of invasive features on cross-sectional imaging, supported the likelihood of a Warthin’s tumor. To put it simply again, [^18^F]FDG PET/CT identified intense metabolic activity, retrospective salivary scintigraphy showed the classic focal uptake with persistent retention suggestive of Warthin’s tumor, brain MRI (despite its screening protocol) and contrast neck CT provided concordant anatomic detail and lacked invasive features. Ultimately, cytologic confirmation via fine-needle aspiration established the diagnosis of Warthin’s tumor. The patient underwent surgery for the primary lung cancer, while the parotid lesion was managed conservatively, given its imaging characteristics, stability on follow-up, and absence of symptoms. The combined cross-modal information increased diagnostic confidence, focused the subsequent FNAC, and helped avoid premature escalation to more aggressive interventions. Warthin’s tumor is known as one of the representative FDG-avid incidentalomas that are discovered incidentally in [^18^F]FDG PET/CT performed for the purpose of diagnosing, staging, or evaluating the treatment of other cancers. However, the frequency of incidentalomas discovered at the level of the parotid gland that are ultimately diagnosed as Warthin’s tumor is relatively low. As for the previous report, the incidence of Warthin’s tumor in [^18^F]FDG PET/CT was 0.87% [12]. In addition, the prevalence of malignancy among parotid incidentalomas has been reported as low in several studies [13,14]. Although the SUV values of Warthin’s tumor and malignant parotid tumors overlapped somewhat, Warthin’s tumor did demonstrate increased radiotracer uptake and persistent retention on salivary gland scintigraphy, and it was reliably distinguished from other parotid gland tumors by the use of salivary gland scintigraphy [15]. The exact mechanism of high FDG accumulation in Warthin’s tumor is not yet known. When high FDG accumulation is detected in the parotid gland, an integrated consideration of the physical examination results, medical history, and ^99m^Tc-pertechnetate scintigraphy enables differentiation between Warthin’s tumor and other lesions [16]. The combined use of FDG PET/CT and salivary scintigraphy is not routinely performed, and data in the literature are limited. However, the complementary information may increase diagnostic confidence in selected situations. PET/CT provides high sensitivity for metabolically active lesions, whereas scintigraphy may demonstrate characteristic uptake and retention patterns in Warthin’s tumor. In cases where FNAC is not feasible or yields non-diagnostic results, combining these modalities could potentially guide clinical decision-making, although pathological confirmation remains the gold standard. In this case, multimodality imaging was crucial in narrowing the differential diagnosis and preventing misinterpretation as metastatic disease or a malignant tumor. In conclusion, this case underscores the importance of correlating [^18^F]FDG PET/CT findings with anatomic imaging (CT, MRI) and past imaging modalities, including salivary gland scintigraphy. Understanding the characteristic imaging features and diagnostic accuracy limits of each modality, as well as obtaining cytologic confirmation when needed, is essential to avoid misinterpretation, prevent unnecessary overtreatment in oncologic patients, and optimize patient management.

## Data Availability

The data that support the findings of this study are available from the corresponding author, M.C., upon reasonable request due to privacy concerns.
